# Evaluation of the Swat River, Northern Pakistan, water quality using multivariate statistical techniques and water quality index (WQI) model

**DOI:** 10.1007/s11356-020-09688-y

**Published:** 2020-07-04

**Authors:** Shah Jehan, Ihsan Ullah, Sardar Khan, Said Muhammad, Seema Anjum Khattak, Tariq Khan

**Affiliations:** 1 National Centre of Excellence in Geology, University of Peshawar, Peshawar, 25130 Pakistan; 2 Department of Environmental Sciences, University of Peshawar, Peshawar, 25120 Pakistan; 3 Department of Environmental Sciences, University of Haripur, Haripur, 26620 Pakistan

**Keywords:** Water quality index (WQI) model, Sodium adsorption ratio, Principal component analysis, Geostatistical techniques, Human health risk assessment, Swat River

## Abstract

This study evaluates the characteristics of water along the Swat River, Northern Pakistan. For this purpose, water samples (*n* = 30) were collected and analyzed for physicochemical parameters including heavy metals (HM). The mean concentrations of physicochemical parameters and HM were within the drinking water guideline values set by the World Health Organization (WHO 2011) except 34%, 60%, and 56% of copper (Cu), nickel (Ni), and lead (Pb), respectively. Pollution sources were identified by various multivariate statistical techniques including correlation analysis (CA) and principal component analysis (PCA) indicating different origins both naturally and anthropogenically. Results of the water quality index (WQI) ranged from 13.58 to 209 with an average value of 77 suggesting poor water quality for drinking and domestic purposes. The poor water quality was mainly related to high sodium (alkalinity) and salinity hazards showing > 27% and 20% water samples have poor alkalinity and salinity hazards, respectively. Hazard quotient (HQ) and hazard index (HI) were used to determine the health risk of HM in the study area. For water-related health risk, HQ_ingestion_, HQ_dermal_, and HI values were > 1, indicating noncarcinogenic health risk (NCR) posed by these HM to the exposed population.

## Introduction

Water is the most limited and countable resource, and only a small fraction (2.5%) of surface water is fresh and suitable for various purposes (household activities, agriculture practices, aquatic biochemical processes, and industrial maneuver) to humans and other living beings (Avci et al. 2018; Xiao et al. 2019). The investigation of water quality and its resources is essential for understanding the current state of water and the main threats that have occurred over the past decades. Natural (i.e., erosion and weathering) and anthropogenic activities have drastically and exceedingly reduced the volume and quality of water globally (Jehan et al. 2019a; Rashid et al. 2019a). Anthropogenic factors are a set of different activities that depend on intensity and time. The quality of water can be damaged either from point or nonpoint sources, and point sources can easily be recognized by their direct discharge into water bodies (Hou et al. 2016; Mukate et al. 2019). Industrial zones and households are the main known point sources that discharge their effluents into surface waters like streams and rivers without treatment (Jehan et al. 2018). Point source is one of the serious problems in developing countries where most commonly people and concerned authorities do not follow the rules and regulations for the environment (Maillard and Santos 2008). Nonpoint sources pertain to a term where no point can exist but mostly surface runoff and agriculture practices are considered (Ekere et al. 2019).

The contamination of water sources has become an alarming issue in developing and developed countries over the last decades (Omonona et al. 2019) due to rapid urbanization and high-level agriculture practices especially in developing countries (Tripathi and Singal 2019). Water quality has a direct connection to humans and wastewater straightaway is used in agriculture sectors (Calazans et al. 2018; Ladwani et al. 2012). The use of municipal wastewater for agriculture is one of the established practices, particularly in urban and peri-urban regions. It is estimated that about 80% municipal wastewater in developing countries is used for crop irrigation (Zeng et al. 2015). Irrigation with wastewater and the use of fertilizers and pesticides greatly affect the physicochemical characteristics and quality of water (Ali et al. 2019; Rashid et al. 2019b).

Numerous studies have been conducted on the assessment of water quality using the water quality index (WQI) model to transform values of different water quality parameters into simple countable numbers so that each and every one can understand the status of water sources. Wu et al. (2018) assessed river water quality using the water quality index model in Lake Taihu Basin, China, suggesting moderate water quality in the basin. Mukate et al. (2019) developed a new integrated water quality index model (IWQI) to evaluate the suitability of groundwater in India. According to their adopted integrated model, drinking water was classified as 2% excellent, 8% poor, 8% unsuitable for drinking purposes, 39% good, and 43% as having marginal characteristics. Similarly, Solangi et al. (2019) evaluated groundwater quality using the WQI in Sujawal district, southern Sindh Province, Pakistan. Their finding revealed 2.13%, 6.38%, 13.83%, 22.34%, and 55.32% of water samples, respectively, were excellent, good, unsuitable for drinking purposes, very poor, and poor.

In Pakistan, surface water quality is deteriorated through industrial discharge, agrofertilizers, and commercial and domestic sewages that released toxic pollutants to the surrounding environment. Usage of wastewater for irrigation is a popular and common practice in all agriculture countries because it supplies nutritional components and reduces expenditure over fertilizers (Lazarova and Bahri 2004). However, the long-term uses of municipal/industrial wastewater for irrigation not only affect the quality of soil but also cause phytotoxicity and human health and environmental problems. Likewise, a big problem associated with the use of such water is the augmenting of high salinity and excessive ammonium ions that leads to the phytotoxic nature of organic wastes which ultimately inhibit seed germination (El Hamouri et al. 1996). In the study area, the Batkhela canal is mainly used for irrigation purposes, and the population is exposed to the water by daily consumption for drinking and bathing. The inhabitants of the study area disposed their solid wastes and commercial and industrial effluents are released directly into the canal water. These anthropogenic activities may create a dangerous situation which needs an immediate attention. Indiscriminate discharge of domestic and industrial wastewater deteriorates the water quality of the study area. No comprehensive scientific studies exist to date about the surface water quality, sources of pollutants, and associated human health risk. Therefore, the present study focused on (1) the impacts of anthropogenic activities on surface water quality, (2) calculation of water quality by performing the water quality index model and sodium adsorption ratio, and (3) elaboration of human health risks for adults and children through ingestion and dermal contact.

## Study area

### Location, climate, and rainfall patterns

**Fig. 1:**
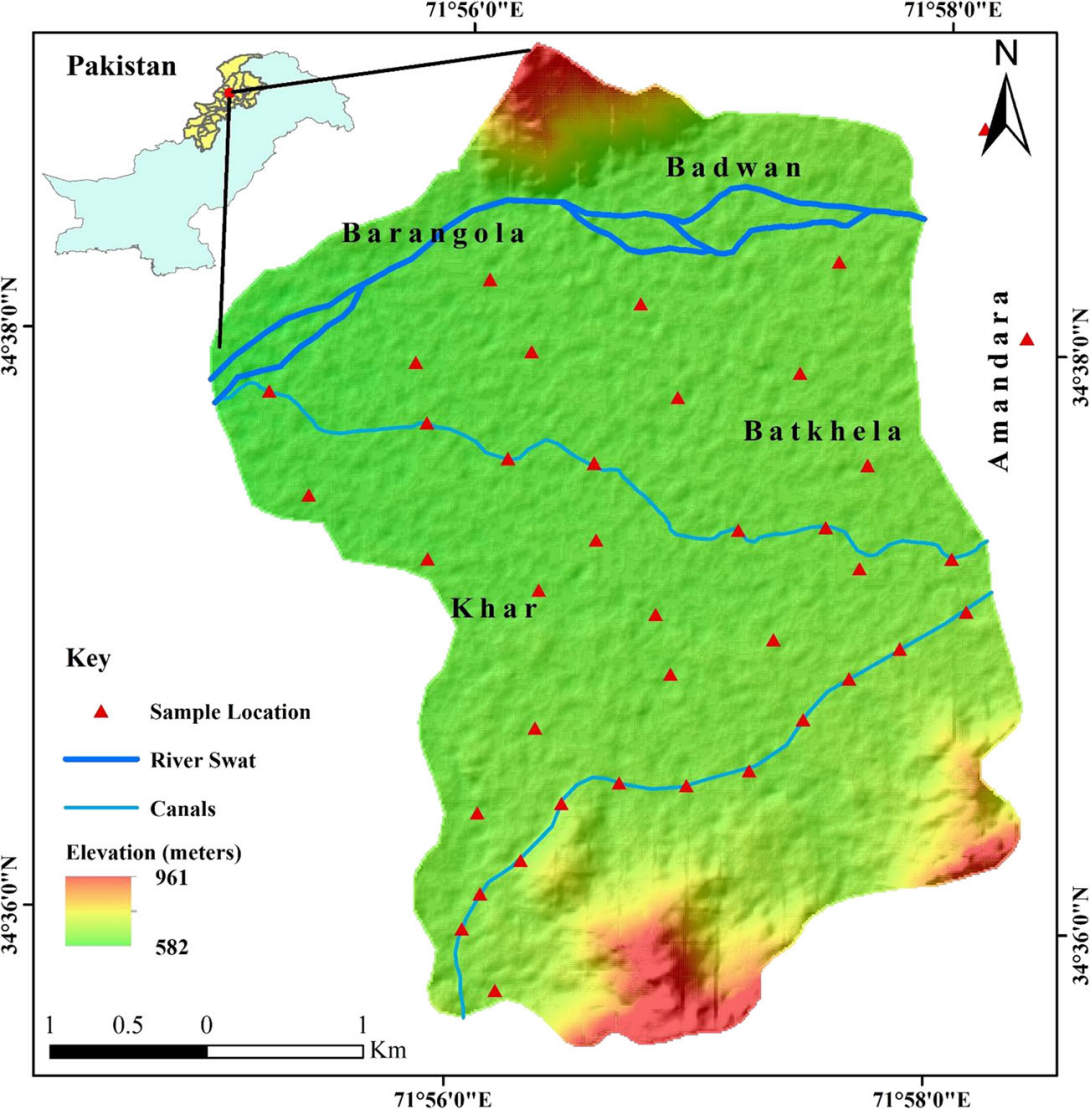
Sampling location map of the study area

The study area (Fig. 1) lies within the geographic coordinates of 34–35° N and 71**–**72° E bounded by the Swat foothills in the north, the Mohmand Melange Complex and the district of Charsadda in the southwest, and the Kot Melange Complex in the west. The study area and its surrounding agricultural fields are recharged and irrigated by the Batkhela canal and its tributaries. Batkhela is a green and lush place surrounded by mountains from all sides and the Swat River is flowing to the west of the city from which a canal was drawn at Amandara for the purpose of irrigation and hydropower. The inhabitants of the study area are mainly associated with agricultural and farming sectors, growing major crops such as rice, maize, wheat, grains, barley, and vegetables. The area is popular for rice production where surface water supplemented with groundwater is used for irrigation. Therefore, the assessment of surface water and human health risk assessment are quite essential along the Swat River, Northern Pakistan.

Climatically subtropical and moist temperate zone dominated the study area with thunderstorm and snowfall (Barinova et al. 2013). The selected area has a hot summer (41.9 °C) and cold winter (0.8 °C). The hottest month is June with mean temperature of 33 °C and and sometimes raised to 46 °C, while the coldest month is January having average temperature of 11 °C to − 2 °C. Winter is longer than summer, and the temperature usually falls below freezing point (November to March). According to the Pakistan Meteorological Department meteorological data, the average annual rainfall is 1003 mm. Maximum rainfall occurs during the months of February (162 mm) and March (231 mm). The average relative humidity varies from a minimum of 40% in April to a maximum of 85% in July (Nafees et al. 2008).

### Soil types, geomorphology, and drainage patterns

Soil plays an important role in finding out the quantity as well as the quality of the water potential in the place. The study area soil is of five major types: Aridisols with limited leaching and water deficiency; Entisols on the unconsolidated parent rock material, on the steep slopes, and along the river course; Gelisols at extremely high elevation which is almost frozen throughout the year; Inceptisols which are found on a fairly steep slope and young geomorphic setting; and Mollisols, the world’s most productive soil, which are at the middle to higher latitude of the study area.

Drainage pattern is an important factor that helps in the determination of potentiality of water (Nasir et al. 2018). The types of drainage and its density describe the surface/subsurface characteristics like runoff, infiltration relief, and permeability. The runoff of an area is characterized by its drainage density as it is the quantum of relative rainwater that might have infiltrated. Hence, the lesser the runoff, the higher the probability of recharge. In the study area, the calculated drainage density is 1.002 km^2^ and the overall drainage pattern shows that drainage density is very high to high along the tributaries of the Swat River. Low to very low drainage was identified along the whole area of subvalleys from north to south in the form of local streams like small tributaries, ephemeral streams, and springs. The distribution of the drainage pattern across the valley is mainly attributed to the weak zones generated by past geological activities (Chuma et al. 2013).

### Hydrology and geological settings

The occurrence and distribution of groundwater is regulated by different hydrological settings in the study area. Regional hydrology describes that how many of inhabitants of the area consumed different groundwater sources. All these water sources mostly receive recharge from precipitation. Shallow aquifers of groundwater have mostly low water table and are used for drinking, domestic, agriculture, and industrial purposes. The inhabitants consume water from groundwater sources like tube wells, hand pumps, dug wells, bore well, and springs. The municipal community tube well water is distributed through supply lines in the study area.

**Fig. 2:**
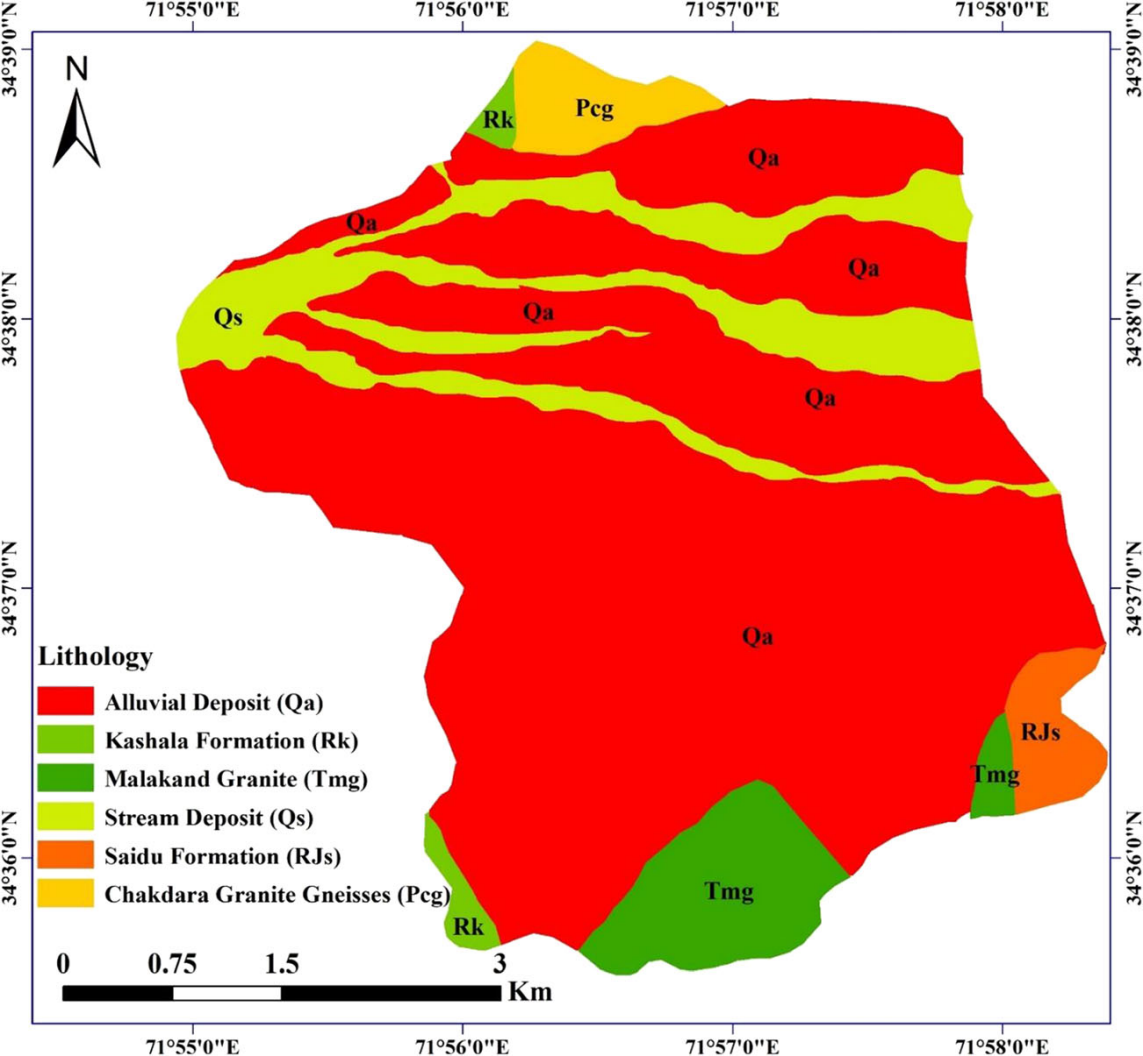
Geological map showing the local setting and different formations of the study area (after Searle and Khan 1996)

The regional geology of the study area includes different formations (Fig. 2), such as Alluvial Deposits (Qa), Kashala Formation (Rk), Malakand Granite (Tmg), Stream Deposit (Qs), Saidu Formation (RJs), Chakdara Granite Gneisses (Pcg), Indus suture mélange, Dir meta sediments, Peshmal Schist, Kohistan batholith, and granite formation. These formations consist of enriched minerals and rocks, i.e., RJs include a combination of graphitic phyllite with garnet grains with thinly laminated marble beds. Rk includes garnet bearing calcareous and graphitic schist, with thin bedded limestone and marble. Tmg is composed of amphibolites, schist, garnet, biotite, schist, and schistose marble, while Pcg contained granite with siliceous schist layer (Nasir et al. 2018; Searle and Khan 1996).

## Material and methods

### Sampling collection and analysis

Thirty water samples (*n* = 30) were collected in replicates from different sites in such a way that each sample shows equal representation of the study area. Samples were collected in 250 mL precleaned polythene bottles which were previously soaked and washed with 10% HNO_3_ after an equal interval distance of 100 m. During sampling collection, pH, electrical conductivity (EC), and total dissolved solids (TDS) were determined in situ by a HANNA HI129 pH meter, conductivity meter (APHA 145), and TDS meter (APHA 208D), respectively. Before further analysis, water samples were kept at 4 °C and immediately transported to the laboratory of the National Centre of Excellence in Geology, University of Peshawar adopting the procedure of Rehman et al. (2018). Latitude and longitude coordinates were also noted for each sampling point using the global positioning system (GPS). Major anions viz. chloride (Cl), sulfate (SO_4_), and bicarbonate alkalinity (HCO_3_) were determined by the trimetric titration method and using UV–visible DR (500) spectrophotometer, respectively. The concentrations of light metals (LM) and HM were analyzed by flame atomic absorption spectrophotometer (FAAS-PEA 700, USA) and graphite furnace atomic absorption spectrometer (GFAAS-PEA 700, USA), respectively. Quality control and assurance were assessed using duplicates, reagent blanks, and certified reference materials (CRM 700), with each batch of water samples. Matrix interference (blank) was < 2% for all elements. Triplicates of sample analysis yielded relative percent differences of < 5%. Each calibration curve was evaluated by analyses of quality control standards before, during, and after the analyses of a set of samples. When the recovery rate becomes out of the recommended range (90–110%), samples were reanalyzed with a new calibration curve.

### Irrigation water quality assessment

Irrigation water quality assessment (IWQA) denotes various mineral composition of water and was primarily developed for the assessment of water quality for irrigation purposes (Abbasnia et al. 2018). The chemical composition of irrigation water quality (IWQ) affects directly or indirectly crop yields and nutrient availability, respectively (Khalid 2019). IWQ was assessed by sodium adsorption ratio (SAR), sodium percentage (Na%), residual sodium carbonate (RSC), magnesium adsorption (MAR), and Kelly ratio (KR) in this study.

SAR for irrigation water quality was evaluated using the following equation adopted by Xiao et al. (2019).

1 $$ \mathrm{SAR}=\mathrm{Na}/{\left[\left(\mathrm{Ca}+\mathrm{Mg}\right)\right]}^{0.5} $$

Four categories were identified for salinity hazard starting from very high to very low as follows: C4 (very high, EC > 2250 μS/cm), C3 (high, 750 < EC < 2250 μS/cm), C2 (medium, 250 < EC < 750 μS/cm), and C1 (low, EC < 250 μS/cm). Likewise, the alkalinity (sodium) hazard was also distributed into four classes starting from very high to low alkalinity hazards: S4 (very high, SAR > 26), S3 (high, 18 < SAR < 26), S2 (medium, 10 < SAR < 18), and S1 (low, SAR < 10) (Raju et al. 2011). Na%, RSC, MAR, and KR are crucial components for IWQA and were determined using the following equations as adopted by Selvakumar et al. (2017).

2 $$ \mathrm{Na}\%=\left(\mathrm{Na}+\mathrm{K}\right)/{\left(\mathrm{Ca}+\mathrm{Mg}+\mathrm{Na}+\mathrm{K}\right)}^{100} $$

3 $$ \mathrm{RSC}=\left[\left({\mathrm{HCO}}_3+{\mathrm{CO}}_3\right)-\left(\mathrm{Ca}+\mathrm{Mg}\right)\right] $$

4 $$ \mathrm{MAR}=\mathrm{Mg}\times 100/\mathrm{Ca}+\mathrm{Mg} $$

5 $$ \mathrm{KR}=\mathrm{Na}/\mathrm{Ca}+\mathrm{Mg} $$

Na% and RSC are categorized into 3 major types for IWQA, i.e., suitable (Na% < 30, RSC < 1.25), marginalized suitable (Na% 30–60, RSC 1.25–2.5), and unsuitable (Na% > 60, RSC > 2.5) (Li et al. 2016). The maximum acceptable level for MAR is 50 meq/L, above which water is considered unsuitable for IWQ (Mahfooz et al. 2019). KR values greater than 3.00 and less than 1.00 are considered unsuitable and suitable for irrigation purposes, respectively (Kelly 1957).

### Water quality index model

The WQI model is an important tool for testing water quality in terms of its potability and management perspective. The WQI model is a water rating scale that reflects the combined influence of a variety of parameters on the overall quality of water (Mohebbi et al. 2013). According to Ekere et al. (2019), WQI was first formulated by Horton (1965) as the numerical procedure to identify water quality from the Ohio River Valley Water Sanitation Commission United States of America. It is very effective in the provision of water quality data in a very simple way to the public, policymakers, and management authorities for making law and legislation for safety water uses. These indices are used for the significant assessment of water quality in various countries of the world (Khalid 2019). The WQI model was computed using the following equation (Eq. 6):

6 $$ \mathrm{WQI}=\sum \left[ Wi\times \left( Ci/ Si\right)\times 100\right] $$

Where *W*_*i*_ = *w*_*i*_/Ʃ*w*_*i*_ is the relative weight, and *w*_*i*_ is the assigned weight ranging from 1 to 5 which is the attribution of each element according to its relative effect on drinking water and human health; 1 and 5 values are assigned to parameters with least to highest critical health effects, respectively. For example, Pb and Cr assigned the highest weight of 5 (Table 1) were considered toxic for health. Ʃ*w*_*i*_ is the weight of the *i*th parameter and is the sum of *w*_*i*_, *S*_*i*_ is the drinking water guideline value (WHO), and *C*_*i*_ is the measured trace element concentration of each element (Khalid 2019). Based on the WQI model, water quality can be divided into 5 categories as follows: (1) (WQI < 50, excellent water quality); (2) (100 < WQI < 200, poor water quality); (3) (200 < WQI < 300, very poor); and when (WQI > 300), water was unsuitable for drinking purposes (Mukate et al. 2019).

**Table 1:** Statistics of physicochemical parameters and the parameters for Water Quality Index calculation

Parameter	Unit	WHO	Descriptive statistics					Parameters for WQI calculation	
			Min	Max	Ave	SD	SE	Weight (wi)	Relative weight (Wi)
pH		6.5–8.5	7.00	8.21	7.23	0.38	0.06	4.00	0.05
EC	μS/cm	1500	103	1260	426	289.25	52.8	4.00	0.05
TDS	mg/L	1000	65.9	806.0	228	189.03	34.5	4.00	0.05
Cl	mg/L	200	35	115	93.01	14.11	1.25	4.00	0.10
SO_4_	mg/L	500	76.0	168	113	19.0	7.83	3.00	0.07
HCO_3_	mg/L	500	116	430	101	23.0	3.91	1.00	0.02
Na	mg/L	200	17.2	39.63	24.3	6.30	1.15	3.00	0.04
K	mg/L	12	2.10	16.31	7.03	3.87	0.70	2.00	0.02
Ca	mg/L	75	27.2	125.2	101	19.44	3.54	2.00	0.02
Mg	mg/L	50	21.2	41.10	26.7	4.77	0.87	2.00	0.02
Zn	μg/L	3000	9.00	74.00	26.3	14.45	2.63	3.00	0.04
Co	μg/L	50	8.00	48.00	22.6	10.48	1.21	1.00	0.01
Cu	μg/L	50	12.0	57.00	32.6	12.72	2.23	2.00	0.03
Ni	μg/L	20	1.00	71.00	27.8	21.01	3.83	4.00	0.05
Pb	μg/L	10	2.00	18.00	9.3	3.43	0.62	5	0.06

### Risk assessment

To assess human health risk of HM in the water compartment of the study area, ingestion through drinking and skin absorption through dermal contact are usually considered (Zeng et al. 2015). The dietary average daily dose (ADD) exposures for direct ingestion (ADD_ingestion_) and dermal absorption (ADD_dermal_) were obtained using Eqs. (7) and (8) (USEPA 2011) shown as follows:

7 $$ {\mathrm{ADD}}_{\mathrm{ingestion}}=\frac{\mathrm{Cw}\times \mathrm{IR}\times \mathrm{EF}\times \mathrm{ED}}{\mathrm{BW}\times \mathrm{AT}} $$

8 $$ {\mathrm{ADD}}_{\mathrm{dermal}}=\frac{\mathrm{Cw}\times \mathrm{SA}\times \mathrm{Kp}\times \mathrm{EF}\times \mathrm{ET}\times \mathrm{ED}}{\mathrm{BW}\times \mathrm{AT}} $$

Where Cw is the average value (μg/L) of HM in the water sample; IR is the daily intake (ingestion) rate (L/day), which was 2.0 and 0.64 for adults and children, respectively (Xiao et al. 2019); SA is the surface skin area (cm^2^), 1800 cm^2^ for adults and 6660 cm^2^ for children; EF is the exposed frequency (days/year), which was taken 350 days/year in this study (Xiao et al. 2019; Zeng et al. 2015); ET is the exposure time (h/day), 0.58 h/day for adults and 1.0 h/day for children (Wang et al. 2011); ED is the exposure duration in years, 70 years for adults and 6 years for children (USEPA 2011); BW is the average body weight (kg), 65 kg and 20 kg for adults and children, respectively (Xiao et al. 2019); and AT is the average time for noncarcinogenic (days); 25,550 days for adults and 2190 days for children (USEPA 2011). Kp is the skin adherence time in the samples (cm/h), 0.0001 cm/h for Pb, 0.001 cm/h for Cu, 0.004 cm/h for Ni and Co, and 0.0006 cm/h for Zn as taken in this study (USEPA 2011). The noncarcinogenic risk (NCR) was calculated by the hazard quotient value (HQ) as shown in Eq. (9); (USEPA 2011). When HQ value > 1, then this predicts potential noncarcinogenic effects.

9 $$ \mathrm{Hazard}\ \mathrm{Quotient}\ \left(\mathrm{HQ}\right)=\mathrm{ADD}/\mathrm{RfD} $$

Where RfD is the reference dose (μg/kg/day) for the different analytes; RfD_dermal_ = RfD × ABS_g_, RfD_dermal_, and RfD_ingestion_ are given in Table 3. ABS_g_ is the gastrointestinal absorption factor, which is unitless. In this study, the RfD values are 3.8% for Cr, 57% for Cu, 4.0% for Ni, 11.7% for Pb, and 20% for Zn (Wang et al. 2017).

Furthermore, the total potential human NCR caused by different pathways was assessed by HI (Eq. 10):

10 $$ \mathrm{Hazard}\ \mathrm{Index}\ \left(\mathrm{HI}\right)=\sum \left({\mathrm{HQ}}_{\mathrm{ing}}+{\mathrm{HQ}}_{\mathrm{derm}}\right) $$

Similarly, if the HI were found > 1, the toxic human health effect should be taken into consideration (USEPA 2011).

### Statistical analysis

Multivariate statistical analysis of the HM concentrations was performed by Pearson’s correlation analysis (CA) and principal component analysis (PCA) using Statistical Package for Social Sciences (SPSS Inc., version 20, Chicago, IL, USA). The Kaiser–Meyer–Olkin (KMO) and Bartlett’s tests were conducted to test the suitability of the data for PCA (Varol 2011). KMO is a measure of sampling accuracy indicating which may be caused by underlying factors. A high value (relatively close to 1) generally indicates that PCA could be useful, as the case in this study, where KMO = 0.36. Bartlett’s test of sphericity shows whether a correlation matrix is an identity matrix, which could indicate the variables are unrelated. The significance level of 0 in this study (less than 0.05) showed that there were significant relationships among the variables. Study area and contour maps of heavy metal distribution were drawn by ArcGIS software (10.2.2).

## Results and discussion

### Water characteristics

**Fig. 3:**
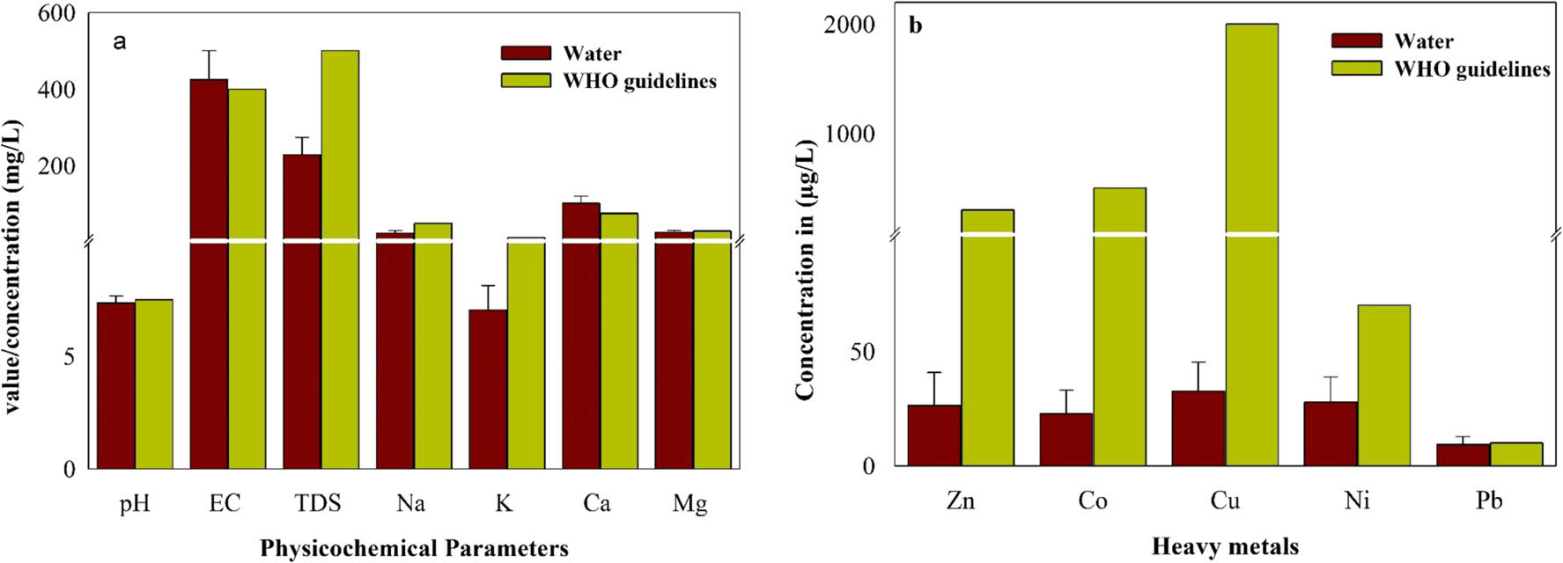
Comparison of **a** physicochemical parameters and **b** heavy metals in water samples of the study area with international standards

Descriptive statistics such as mean, minimum, maximum, standard deviation, and standard error of water quality parameters were performed and compared with the WHO (2011) drinking water guidelines (Table 1; Fig. 3). Water shows a slightly alkaline nature with average pH value of 7.34. The pH value in 30% of the samples exceeded the WHO (2011) drinking water guideline value, which can affect the mucous membrane (Xiao et al. 2019). Both EC and TDS values were found lower than the WHO (2011) guideline (Fig. 3a). Chloride is a significant major anion that is released from both geogenic and anthropogenic sources like weathering, erosion, atmospheric deposition, agrofertilizers, and effluents/leachates from industries. The mean Cl concentration (93.01 mg/L) was found lower than the recommended (200 mg/L) WHO health guideline. A high concentration of Cl in drinking water does not pose toxicity to humans, but it imparts a detectable salty taste in water above 200 mg/L concentration (Samantara et al. 2017). The mean values of SO_4_ and bicarbonate HCO_3_ were 113 and 101 mg/L, respectively, which were below the WHO health guideline (500) value as shown in Table 1. Both anions do not pose any significant health risk; however; their high concentration than normal deteriorates water quality causing problem which may risk our health. The dominance of anions was in the order of Cl > HCO_3_ > SO_4_. The average concentrations of all LM (except Ca which exceeded in 90% of the water samples) were within the permissible limits of drinking water guideline values set by the WHO (2011) (Fig. 3a). Both Ca and Mg are important micronutrients which are essential for the human body and mainly originate from dissolution of CO_3_ minerals and dolomitic rocks, respectively. Na and K generally originate from dissolution of clay minerals and evaporates and silicates (Avci et al. 2018). The order of abundance of LM is Ca > Mg > Na > K, which is in accordance with the study conducted by Sánchez et al. (2017) in water–rock interactions in the urban area of Puebla Valley aquifer (Mexico). The mean concentration of all HM was found lower except in 34%, 60%, and 56% of Cu, Ni, and Pb, respectively (Fig. 3b). Among HM, Pb is one of the most toxic and carcinogenic metals that enter into water bodies from various anthropogenic sources via household, paint, vehicles, and industrial emission (Arain et al. 2009). Pb has potential geogenic mafic and ultramafic sources already assessed by many researchers in the study area (Khan et al. 2018; Liu et al. 2020; Rashid et al. 2018). Its toxicity can be seen in a little amount in humans causing disorders in the human nervous system and affecting the digestive as well as the skeletal system. The mean concentration of Pb was found higher than that in the studies by Ashraf et al. (1991) in water from three freshwater reservoirs on the Indus River, Pakistan, and Ilyas and Sarwar (2003) in drinking water samples in the vicinity of Palosai drains, Peshawar. Similarly, Ni is a significant toxic HM causing various diseases such as allergy, dermatitis problems, and kidney disorders, and chronic exposure is associated with cancer risk. The mean concentration of Ni was found higher than those reported by Haq (2005) in surface and groundwater contamination in NWFP (former name of Khyber Pakhtunkhwa) and Sindh provinces, Pakistan. Anthropogenic activities like agriculture and the industries in the surrounding vicinity of streams, rivers, and canals can contaminate water severely and such contamination becomes serious in densely populated areas especially in unplanned urban cities (Rehman et al. 2008). The concentration of HM was greater compared with light metals in all the studied water samples (Fig. 3a, b) in the present study.

### Hydrochemical facies of the study area

**Fig. 4:**
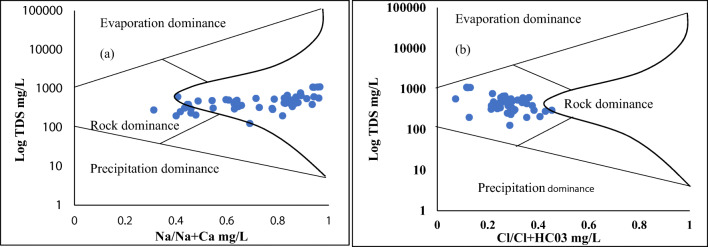
**a**, **b** Schematic diagram to understand the major ion chemistry and mechanism controlling water chemistry along the Swat River, Pakistan. Data were plotted as Na/Na + Ca mg/L versus Log TDS and Cl/Cl + HCO_3_ mg/L versus Log TDS

The Gibbs (1970) diagram is useful for the valuation and estimation of different mechanisms that control the hydrogeochemistry of an area mainly influenced by three components: precipitation, weathering of parent rocks, and evaporation. The water composition is controlled by ambient climatic conditions, groundwater movement, and mineral features. Therefore, for evaluation of hydrochemical features and groundwater interactions, the Gibbs diagram was designed to establish a connection between the aquifer’s geology and water composition (Gibbs 1970). For understanding the mechanism for hydrogeochemical control, water data was arranged by plotting total dissolved concentrations (TD) versus anion combination of (Cl^−^/Cl^−^ + HCO_3_^−^) and weight ratio of cation concentrations (Na^+^/Na^+^ + Ca^++^) (Gibbs 1970; Fig. 4 a and b). The water type of the study area along the Swat River, Northern Pakistan, mostly falls into a rock-dominant region admitting that surrounding rocks previously discussed in study geology have a great influence on the water data. Thus, the Gibbs diagram (Fig. 4 a and b) deciphered that weathering of parent rocks and regional hydrogeological settings play a key role in water composition and chemistry. Further, some of the water features next to rock dominancy fall into recharge and cation exchange mechanism that make a suitable proportion for water composition in the study area.

**Fig. 5:**
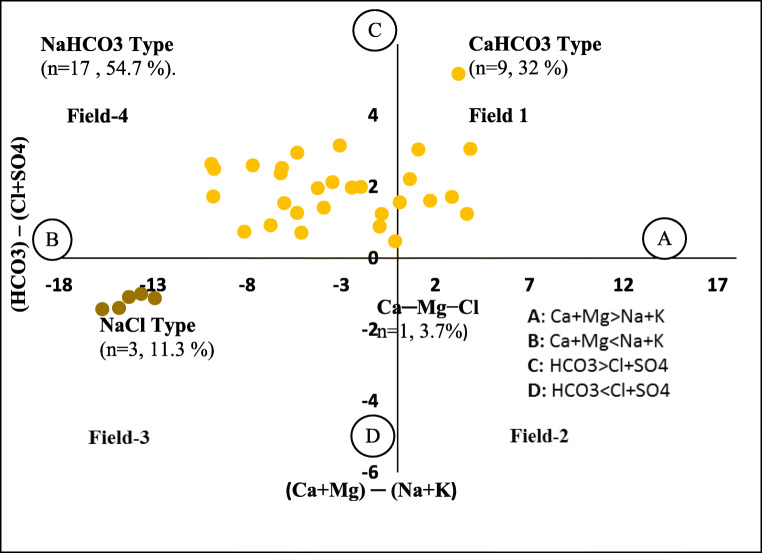
The Chadha diagram identifies different water type formations along the Swat River, northern Pakistan. After Chadha (1999). Field 1: Ca–HCO_3_ type water indices, representing both recharge and weathering processes. Field 2: Ca–Mg–Cl type waters, reflecting reverse ion-exchange processes. Field 3: Na–Cl type waters, indicating evaporation is the principal mechanism. Field 4: Na–HCO_3_ type waters, reflecting cationic and ionic exchange processes

Chadha (1999) proposed the hydrochemical characteristic for the classification of water data by plotting data focused on the differences of cations and anions in terms of presenting % [HCO_3_ and (Cl + SO_4_)] concentrations in meq/L as percentage versus (Ca + Mg) and (Na + K) concentrations in meq/L (Chadha 1999). The resulting figure (Fig. 5) has four fields which described different types of hydrogeochemical processes responsible for water type formations. More than half (*n* = 17) of the samples had NaHCO_3_ water type, *n* = 9 water samples were CaHCO_3_ water type, whereas *n* = 3 water samples comprised the Ca–Mg–Cl water type, and the remaining water samples (*n* = 1) fell in the category of NaCl type (Fig. 5).

### Water quality assessment

The water quality assessment was composed of three components: (1) comparison of HM concentrations with water quality standard (WHO), (2) assessment of water quality for irrigation purposes, and (3) evaluation of water quality using WQI. The HM concentrations were above the drinking water guideline value set by the WHO (2011) in 33%, 55%, and 65% of the samples for Cu, Ni, and Pb, respectively, indicating contamination and undrinkable status of water quality. The main pollution elements were Cu, Ni, and Pb in the study area.

SAR value ranged from 10.72 to 20.67 with a mean value of 13.51 (Table 2). According to sodium hazard, SAR values > 9 were regarded as unsuitable for irrigation purpose (Khalid 2019). In view of the current result, 73% of the samples were found in good quality, while 27% of the water samples were of acceptable quality as shown in Table 2. High SAR values create sodium salinity hazards by reducing the ratio of calcium and magnesium which cause infertility and retardation in plants and crops. In addition, Na% and RSC showed IWQ suitable to marginal suitable except in 23% and 29% of the samples, respectively. Higher RSC value in the study area shows excess dissolution of carbonates in addition to alkaline earth elements (AEE) that render the water unsuitable for irrigation purposes in terms of RSC. MAR values in all the sampling points were recorded above 50 meq/L which is in good agreement with the study conducted by Mahfooz et al. (2019). KR value ranges from 21.4 to 41.3 meq/L in all sampling sites suggesting water unsuitable for irrigation purposes.

**Table 2:** Classification of water quality for irrigation purposes in the study area

Water class	Salinity hazards		Alkali hazards	
	EC (μS/cm)	Number of samples (%)	SAR (epm)	Number of samples (%)
Excellent	Up to 250	24	Up to 10	–
Good	250–750	56	10 to 18	73
Fair/medium	750–2250	20	18 to 26	27
Poor/bad	2250–5000		> 26	–

WQI was applied to get a more comprehensive understanding of the water quality in the subdistrict of Batkhela along the Swat River, Pakistan (Table 1). Results of the current study illustrated that WQI ranged from 13.58 to 209 with an average value of 77. According to the water quality index, the water quality of the study area was poor for drinking and domestic purposes. This could be attributed to the presence of a high concentration of HM and variation in some of the other physicochemical parameters due to anthropogenic and geological sources. Anthropogenic activities like agriculture and the industries in the surrounding vicinity of streams, rivers, and canals can contaminate water severely and such contamination becomes serious in densely populated areas especially in unplanned urban cities (Rehman et al. 2008). The results of our study are in good agreement with the study conducted by Khalid (2019) in three districts of Baluchistan Province, Pakistan, and by Xiao et al. (2019) on water quality and health risk assessment of trace metals in river and well water in the Chinese Loess Plateau. They concluded that the major proportion of their study possessed very poor water quality.

### Human health risk assessment

Table 3 shows HQ and HI values for water quality of Batkhela subdistrict along the Swat River, Pakistan. According to the health risk assessment model recommended by the USEPA, the NCR to personal health of the HM in water were calculated. Two population groups comprised of adults and children were considered. For water-related health risk, HQ_ingestion_, HQ_dermal_, and HI (HQ_ingestion_ + HQ_dermal_) were classified as low to high indicating moderate to significant potential hazards. The HQ_ingestion_ values ranged from 0.002 to 0.195 for adults and 0.002 to 0.203 for children. These values indicated that the ingestion exposure had no adverse health effect on the human body and posed no potential noncarcinogenic risk. The HQ_dermal_ values of the toxic HM in adults and children were > 1 (adults 0.004 to 0.317, children 0.016 to 1.77), indicating the calculated HM presented significant hazard through dermal absorption specially to children. The calculated HQ_ingesiton_, HQ_dermal_, and HI values for children were observed to be higher than those for adults. The order of HI in drinking water was Ni > Pb > Co > Cu > Zn in children and Co > Ni > Pb > Cu > Zn in adults. The results of the present study are in good agreement with the study conducted by Mahfooz et al. (2019) who stated that the HM posed hazard to the inhabitants causing different diseases such as stomach and lung cancer, hypertension, anemia, physiological disabilities, and cardiac arrest. The findings of our study were found higher than those reported by Meng et al. (2016) who assessed Dan River drainage (China) water samples for human health risk through HQ by ingestion and HQ by skin. The calculated result in our study through HQ_dermal_ was found above unity for Ni and Co as shown in Table 3.

**Table 3:** Reference dose (RfD), hazard quotient (HQ), and hazard index (HI) for each heavy metal

HM	RfD_ingestion_	RfD_dermal_	HQ_ingestion_		HQ_dermal_		HI = ƩHQs	
			Adult	Child	Adult	child	Adult	Child
Zn	300	60	0.00259	0.00269	0.00405	0.0168	0.00664	0.0168
Co	5	0.06	0.10995	0.11435	0.26655	4.75992	0.3765	1.75992
Cu	40	12	0.02406	0.02503	0.04187	0.26043	0.06593	0.26043
Ni	20	5.4	0.04106	0.0427	0.31754	1.77752	0.3586	1.77752
Pb	1.4	0.42	0.196	0.20384	0.0341	0.70705	0.2301	0.70705

### Statistical analysis

The correlation among HM and physicochemical parameters can provide useful information on the sources and the emission pathways. Correlation matrix was calculated by the Pearson’s correlation coefficients for the elements and the results are shown in Table 4. The correlation constant (*r*) value identifies how significantly and conveniently the water samples are arranged in a simple and straight line. A number of significant positive correlations existed among the selected elements. The element/parameter pairs pH–Mg, EC–TDS, and Na–Mg had a significant positive correlation at < 0.01 significance level, and the element pairs Zn–Ni and K–Mg had a significantly positive correlation at < 0.05 significance level showing that the elements were discharged from the same sources. The main sources of these metals in the water may be from the application of inorganic and nitrogenous and phosphate fertilizers and from animal manures. The combustion of coal and other fuels such as wood, rubber, tires, and other plastic wastes in brick kilns is one of the responsible sources for the emission of HM into the surrounding ambient environment (Khalid 2019).

**Table 4:** Pearson correlation matrix of heavy metals in the study area

HM	pH	EC	TDS	Na	K	Ca	Mg	Zn	Co	Cu	Ni	Pb
pH	*1.00*	− 0.24	− 0.06	− 0.13	− 0.34	0.12	− 0.41^*^	− 0.29	− 0.16	0.105	0.10	− 0.13
EC	− 0.24	*1.00*	0.50^**^	0.31	0.14	0.10	0.031	0.10	− 0.06	− 0.18	− 0.02	− 0.16
TDS	− 0.06	0.50^**^	*1.00*	− 0.03	0.11	− 0.20	− 0.05	− 0.16	− 0.20	− 0.25	− 0.02	− 0.08
Na	− 0.13	0.31	− 0.03	*1.00*	0.24	0.21	0.49^**^	0.27	− 0.00	0.09	0.31	− 0.4^**^
K	− 0.34	0.14	0.11	0.24	*1.00*	0.06	0.45^*^	− 0.03	0.29	0.04	− 0.12	− 0.32
Ca	0.12	0.10	− 0.20	0.21	0.06	*1.00*	0.22	0.25	0.06	− 0.10	− 0.01	− 0.41^*^
Mg	0.41^*^	0.03	− 0.05	0.49^**^	0.45^**^	0.22	*1.00*	0.23	0.21	− 0.04	− 0.03	− 0.29
Zn	− 0.29	0.10	− 0.16	0.27	− 0.03	0.250	0.23	*1.00*	0.11	0.16	0.40^*^	− 0.05
Co	− 0.16	− 0.06	− 0.20	− 0.03	0.29	0.06	0.22	0.11	*1.00*	0.09	0.21	− 0.11
Cu	0.10	− 0.18	− 0.25	0.09	0.04	− 0.10	− 0.04	0.16	0.09	*1.00*	0.25	0.03
Ni	0.10	− 0.02	− 0.02	0.31	− 0.12	− 0.01	− 0.03	0.40^*^	0.21	0.25	*1.00*	0.15
Pb	− 0.13	− 0.16	− 0.08	0.47^**^	− 0.32	0.41^*^	− 0.29	− 0.05	− 0.12	0.03	0.15	*1.00*

^**^ Correlation is significant at the 0.01 level (2-tailed). ^*^ Correlation is significant at the 0.05 level (2-tailed)

Values of dominant parameter in each factor are dominated in bold. Italic data are given for the same parameter correlation with each othe

The PCA was applied to identify the origins of HM in water samples. PCA aims to reduce the dimensionality of a multivariate dataset into fewer components which describes and explains most of the information contained in the data. The KMO value for water was 0.364, and the significance of Bartlett’s sphericity test was 0.01, indicating that the PCA was effective for the studies (Varol 2011). PCA of the whole dataset contributed a maximum of five (5) components responsible for 72.02% of the total variance having eigenvalue (*λ*) > 1.0 (Table 5). The first component (varifactor) of the PCA was responsible for 17.41% of the total variance correlated (loading > 0.60) with Na, Mg, and K. VF1 shows the strength of the LM suggesting that sodium, magnesium, and potassium have a dominant role in factor loading contribution. The second varifactor (VF2) explained 16.07% of the total variance mainly participated by Cu and Ni which strongly correlated with high loadings (> 0.50). It was noteworthy that the high contents of these metals could be related to anthropogenic activities such as industrial processes and agrofertilizer application and commercial and electroplating industries (Gao et al. 2019). The 3rd varifactor (VF3) identifies the influence of Pb and their loading value was observed to be greater than 0.56 indicating a mixed source of natural and anthropogenic sources that influence the water quality in the study area. The 4th and 5th varifactors (VF4 and VF5) show significant loading for Ni (> 0.64) and Cu (> 0.46), respectively. These factors can promote the dissolution of Cu and Ni minerals in the water aquifers in the study area.

**Table 5:** Principal component analysis via dimension reduction method after varimax rotation

HM	F1	F2	F3	F4	F5
pH	− 0.45	0.18	− 0.67	− 0.02	0.39
EC	0.37	− 0.54	− 0.02	*0.53*	0.02
TDS	0.02	− 0.70	0.07	0.43	0.28
Na	*0.73*	0.10	− 0.23	0.26	0.18
K	*0.60*	− 0.22	0.30	− 0.37	0.33
Ca	0.39	0.12	− 0.64	− 0.10	− 0.38
Mg	*0.76*	0.04	0.14	− 0.26	− 0.14
Zn	0.45	0.46	0.08	0.42	− 0.40
Co	0.32	0.32	0.36	− 0.29	0.21
Cu	0.01	*0.58*	0.09	0.05	*0.46*
Ni	0.13	*0.57*	0.08	*0.60*	0.23
Pb	− 0.58	0.15	*0.56*	0.22	− 0.26
Eigenvalue	2.09	1.93	1.77	1.77	1.09
Variability %	17.4	16.0	14.7	14.7	9.15
Cumulative %	17.4	33.4	48.2	63.0	72.2

Values of dominant parameters in each factor are represented in italic

### Spatial distribution mapping

**Fig. 6:**
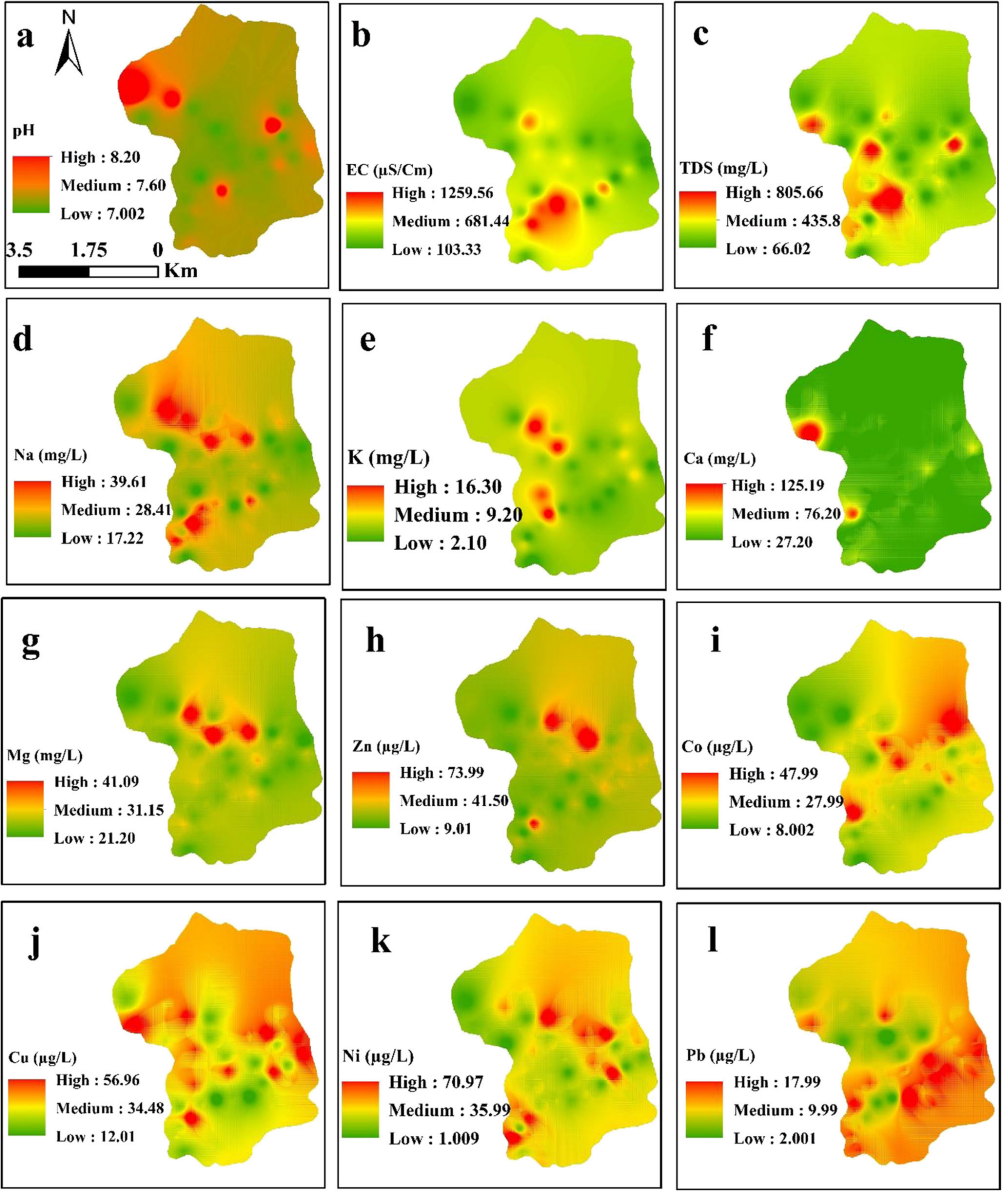
Spatial distribution maps of physicochemical parameters and heavy metals in water samples

Spatial distribution pattern maps of the physicochemical parameters and HM for water samples were drawn using interpolation and kriging techniques which are easily and understandable geostatistical approaches for spatial distribution pattern maps in water (Rashid et al. 2019a). Thus, pH, EC, TDS, Na, K, Ca, Mg, Zn, Co, Cu, Ni, and Zn were mapped using spatial interpolation kriging techniques. Different classes were assigned representing low, medium, and high concentrations in the study area. The spatial variation of the physicochemical parameters and HM in water samples of the study area is shown in Fig. 6. pH is spatially distributed in the water samples of the study area as shown in Fig. 4a. pH was found higher in 30% of the water samples than the recommended guideline values. Although pH affects palatability and has no direct health impacts, it is an important operational water quality parameter. It can affect the corrosivity power of water. At pH above 8, the disinfecting tendency of chlorine decreases and its corrosiveness increases below pH 7 (Ahmed et al. 2015).

Similarly, EC and TDS are spatially distributed and three different classes have been obtained on the basis of its concentration as shown in Fig. 6 b and c, respectively. The three classes are green (low concentration), yellow (medium concentration), and red (highest concentration). The measurement of EC is related to the concentration of ionized substances in water and may be related to the excessive hardness and mineral contamination. In case of TDS, about 75% was found within the desirable limit of 500 mg/L, while 25% of the water samples were found above the desirable limit of 1000 mg/L. Water containing TDS more than 500 mg/L causes gastrointestinal irritation (Jehan et al. 2019b). The concentration ranges for LM like Na and K were 17.22 to 39.61 mg/L and 2.10 to 16.30 mg/L, respectively, in the study area (Fig. 6d, e). The concentration of Na was quite low in the study area, while in 34% of the water samples, K content was found higher than the maximum allowable limit of 12 mg/L (WHO 2011). The main sources of K in water include weathering of potash silicate minerals, the use of potash fertilizers and rain water, and the use of surface water for irrigation. Similarly, three categories were classified for the concentration of Ca and Mg which ranged from 27.20 to 125 mg/L and 21.20 to 41.09 mg/L, respectively (Fig. 6f, g). The maximum desirable limit for Ca and Mg is 75 and 50 mg/L, respectively. In water samples of the study area, Ca content slightly exceeds the Mg concentration in accordance with their relative abundance in the host rocks. The spatial variation of Ca (Fig. 6f) shows that the area is exceeding the desirable limit (< 75 mg/L) and surpassed the permissible limits (75–200 mg/L) which correspond to 90% and 10%, respectively. The spatial variation of Mg (Fig. 6g) shows that in the entire study area, the water samples fall in the desirable limit (50 mg/L) during the study.

Zinc is spatially distributed in the water samples of the study area (Fig. 6h). The 9.01 value represents the minimum concentration and 73.99 indicates highest concentration falling in the desirable limit of 3000 for zinc. The entire water samples in the study area were within the maximum desirable limit of 3000 μg/L set by the WHO (2011). Similarly, there are two classes for Co—lowest to highest—ranging from 8.00 to 48.00 μg/L in the study area (Fig. 6i). The distribution map of Cu at different classes (12.01 to 57. 00 μg/L) is shown in Fig. 6j. The desirable limit for Cu is 50 μg/L. In water samples of the study area, Cu exceeds in 34% of the total analyzed samples. Cu is considered an essential micronutrient, but at high concentration, it can cause liver and kidney failure, nausea, vomiting, and gastrointestinal problems. The distribution pattern map of Ni is shown in Fig. 6k. Two classes differentiate the highest to lowest concentration of Ni ranging from 1.00 to 71.97 μg/L in the study area. The maximum desirable limit set by the WHO (2011) for Ni is 20 μg/L; 60% of the samples were above the recommended desirable limit. Ni content can induce nasal and lung problems when ingested at a higher dose. There are two classes (lowest and highest) for Pb concentration that show the Pb level in the water samples of the study area. The lowest (2.00 μg/L) and highest (17.98 μg/L) concentrations of Pb were recorded in the study area (Fig. 6l). The maximum desirable limit of 10 μg/L was set by the WHO (2011); 56% of the samples were observed to be higher than the desirable limit. The distribution pattern of HM showed that water in the study area is contaminated with Ni, Cu, and Pb; therefore, it is recommended that local residents should use safe groundwater sources for drinking/domestic and bathing needs.

## Conclusion

The present research work thoroughly and briefly describes the water quality and human health risk along the Swat River, Northern Pakistan. Results indicated that large variations of HM in water sample were found in the study area. Among the investigated HM viz. Ni, Cu, and Pb originated from anthropogenic inputs mainly from industrial and agricultural practices and weathering of bed rocks. The poor water quality in this area was mainly related to high alkalinity and salinity hazard. The WQI model also showed that water quality of the study area was found to be unsuitable for drinking and domestic purposes. Similarly, water quality for irrigation in terms of the quality indicator such as SAR was not suitable for irrigation. The noncarcinogenic risk assessment indicated that there was NCR to humans through ingestion and dermal absorption (i.e., HQ_ingestion_, HQ_dermal_, and HI > 1). Children were more sensitive than adults to the noncarcinogenic hazards, based on the HQ and HI value for children and adults. The results of multivariate statistical analysis showed that the water of the study area was greatly influenced by anthropogenic activities such as agricultural and industrial processes and commercial wastewater emission from the surrounding populated city of Batkhela. Therefore, to ensure public health and safety, it is necessary to make regular monitoring assessment of exposure to HM in the district of Batkhela, Pakistan.
